# Duration of Untreated Psychosis and Brain Function during Verbal Fluency Testing in First-Episode Schizophrenia: A Near-Infrared Spectroscopy Study

**DOI:** 10.1038/srep18069

**Published:** 2015-12-10

**Authors:** Po-Han Chou, Wei-Hao Lin, Chih-Chien Lin, Po-Hsun Hou, Wan-Rung Li, Chia-Chun Hung, Ching-Po Lin, Tsuo-Hung Lan, Chin-Hong Chan

**Affiliations:** 1 Department of Psychiatry, Taichung Veterans General Hospital, Taichung, Taiwan; 2 Department of Psychiatry, Faculty of Medicine, National Yang-Ming University, Taipei, Taiwan; 3 Department of Social Work and Child Welfare, Providence University, Taichung, Taiwan; 4 Brain Connectivity Lab, Institute of Neuroscience, National Yang-Ming University, Taipei, Taiwan; 5 Institute of Brain Science, National Yang-Ming University, Taipei, Taiwan; 6 Department of Psychiatry, Conde S. Januário General Hospital, Macau, China

## Abstract

A longer duration of untreated psychosis (DUP) has been associated with poor clinical outcomes in patients with schizophrenia (SZ); however, it remains unclear whether this is due to neurotoxic effects of psychosis. The purpose of this study was to use near-infrared spectroscopy (NIRS) to investigate the influence of DUP on brain function using two verbal fluency tests (VFTs) in patients with first-episode SZ (FES). A total of 28 FES patients and 29 healthy controls (HC) underwent NIRS during VFTs. Group comparisons of cortical activity were made using two-tailed t-tests and the false discovery rate method. We then examined the associations between DUP and hemodynamic changes in each channel to identify any effects of DUP on brain cortical activity. During the letter VFT, the HC group exhibited significantly greater cortical activations over bilateral frontotemporal regions compared to FES patients. However, this distinction was not observed while performing a category version of the VFT. In addition, no associations between DUP and brain cortical activity were observed in the FES group during either VFT. In conclusion, we did not find an association between DUP and frontotemporal cortical activities. This might be because neurodevelopmental disturbances result in neurocognitive deficits long before psychotic symptoms onset.

Duration of untreated psychosis (DUP) is defined as the time from the first manifestations of psychotic symptoms to the beginning of antipsychotic treatment^1^. Longer DUP is associated with poor clinical and functional outcomes^1^,^2^, but the mechanism underlying this relationship is unknown. The neurotoxicity hypothesis by Wyatt in 1991 proposed that periods of untreated psychosis are ‘biologically toxic’ to the brain^3^. This idea was a major impetus for programs designed to shorten DUP to prevent further brain tissue loss or cognitive decline in patients with schizophrenia (SZ) and has also been an important part of rationale for developing early intervention services.

In the past two decades, many studies have investigated the effects of DUP on the brain, but they have yielded conflicting results. Some studies showed a deteriorating effect of DUP on various domains of cognitive functions in patients with first-episode SZ (FES)^4^,^5^,^6^, but most others did not observe negative effects^7^,^8^,^9^,^10^,^11^,^12^. Magnetic resonance imaging (MRI) studies focusing on the issue also reported inconsistent results. Some studies demonstrated that longer DUP had negative effects on brain morphological changes^13^,^14^,^15^,^16^,^17^, but others did not^18^,^19^,^20^,^21^,^22^,^23^.

More recently, the ‘neurotoxicity’ concept was questioned by several groups. A critical review by Rund *et al.* concluded that there was limited evidence for a relationship between DUP and changes in neurocognitive functions or brain structures^24^. Similarly, Anderson *et al.*’s review listed minimal evidence of an association between untreated psychosis and brain structure^25^. These neuroimaging and neuropsychological findings may raise the question ‘Is active psychosis neurotoxic?’ In 2006, McGlashan argued that if active psychosis was indeed neurotoxic, certain manifestations would be expected in the course of the illness, but there is little evidence for such a process^26^.

On the other hand, relatively few functional neuroimaging analyses have addressed this issue. Two studies performed functional MRI (fMRI) to examine the effects of DUP on resting state brain connectivity in drug-naïve FES patients, but they did not find any effects of DUP^27^,^28^. One study used near infrared spectroscopy (NIRS) and found negative effects of DUP on cortical activity during a letter version of the verbal fluency test (VFT) in SZ patients treated with antipsychotics for more than 6 months^29^. However, the study participants were heterogeneous and were in early and chronic stages of SZ; therefore, some confounding factors cannot be totally excluded (i.e. antipsychotic treatment duration or relapsed psychotic episodes).

Uncertainty remains as to whether active psychotic symptoms exacerbate brain cortical activities in patients with SZ. Limiting enrolment to FES patients avoids confounding by illness duration, longstanding substance abuse, and treatment effects^30^. This type of design may provide more generalizable results regarding the nature of the disorder than studies of chronic patients^19^. The aim of this study was to use NIRS to investigate the relationship between DUP and cortical activity over bilateral frontotemporal regions during VFTs to elucidate the effects of untreated psychosis on brain function. There are two versions of VFT based on the type of cue; the category fluency task (CFT) requires the subject to generate words belonging to a specific semantic category, while the letter fluency task (LFT) requires the generation of words based on phonemic cues. A previous NIRS study investigating DUP effects adopted the LFT as a cognitive activation task^29^, but another recent report indicated that CFT performance was one of the candidates to identify SZ endophenotype^31^. Therefore, we employed both VFTs to investigate cortical activity in FES patients. We hypothesised that if psychosis is biologically toxic, longer DUP would be associated with poorer cortical activity during VFTs.

## Results

### Study participant characteristics

The study participants’ demographic characteristics are presented in Table 1. There were no significant differences between the healthy control (HC) and FES groups regarding age, sex, education, or socioeconomic status (SES). For both the LFT and CFT, the HC group performed significantly better than the FES group (LFT; *P* = 0.004, CFT; *P* = 0.004). LFT performance was significantly lower than that of the CFT in HC (*P* = 0.001) and FES groups (*P* = 0.001).

### Cortical activity during the VFT

**Figure 1:**
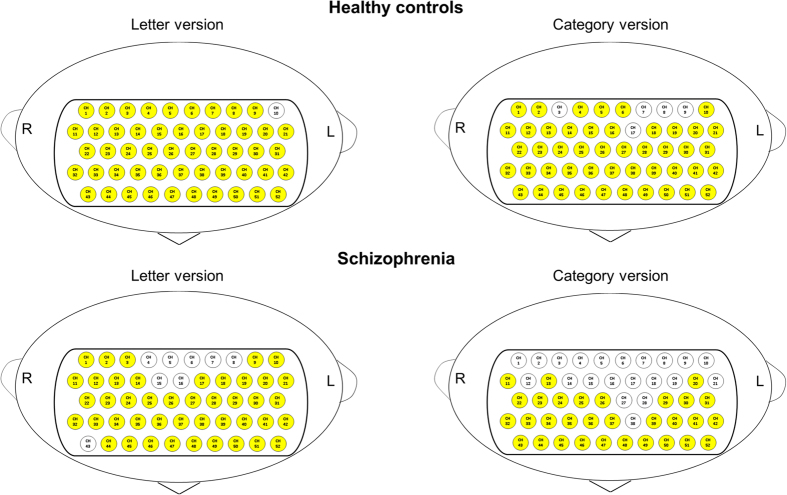
Significant mean [oxy-Hb] changes during the task relative to the pre-task baseline for the letter (LFT, left side) and category (CFT, right side) versions of the VFT in healthy controls (upper) and in patients with SZ (lower).

**Figure 2:**
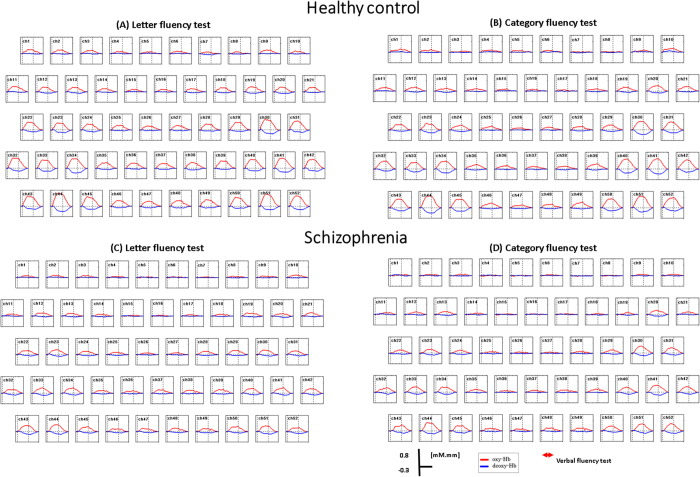
Grand average waveforms. (**A**) LFT in healthy controls. (**B**) CFT in healthy controls. (**C**) LFT in patients with schizophrenia. (**D**) CFT in patients with schizophrenia. Mean [Oxy-Hb] and [deoxy-Hb] changes during VFTs are presented as grand average waveforms in 52 channels in red and blue lines, respectively.

As shown in Fig. 1, there were significant increases in [oxy-Hb] changes during the LFT relative to the pre-task baseline over bilateral frontotemporal regions at 51 channels (except ch10; FDR-corrected, P = 0.000 ~ 0.021) in the HC group and 44 channels (ch1-3, ch9-14, ch17-42, ch44-52; FDR- corrected, P = 0.000 ~ 0.037) in the FES group. With regard to CFT, there were significant increases in [oxy-Hb] changes during the task period at 47 channels (ch1-2, ch4-6, ch10-16, ch18-52; FDR-corrected, P = 0.000 ~ 0.030) in the HC group and 31 channels (ch11, ch13, ch20, ch22-26, ch29-37, ch39-52; FDR-corrected, P = 0.000 ~ 0.029) in the FES group (see Supplementary Table S1, S2, S3, and S4 online). The grand averaged waveforms of [oxy-Hb] and [deoxy-Hb] changes during two types of VFTs in both groups are shown in Fig. 2.

### Group comparison of cortical activity during the VFT

**Figure 3:**
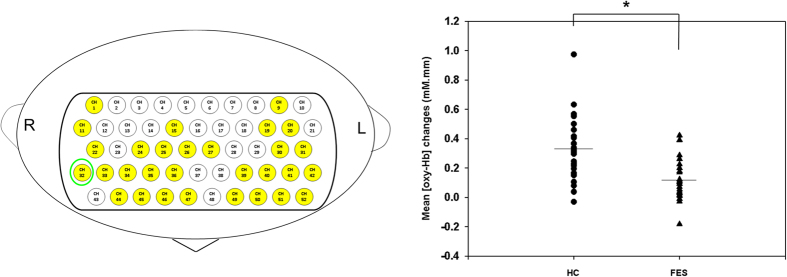
Topographic maps of different hemodynamic response patterns during LFT over bilateral frontotemporal regions. Left: Topographic maps showing clusters of channels with significantly higher cortical activities in the HC group. Right: Dot plots of the mean [oxy-Hb] changes in typical channels during the LFT period. Bars show the averages of the [oxy-Hb] changes at ch32, and asterisks show significant differences between groups (FDR-corrected *P* < 0.05).

Compared with the HC group, the FES group demonstrated significantly lower cortical activity in 30 channels during the LFT (ch1, ch9, ch11, ch15, ch19-20, ch22, ch24-27, ch30-36, ch39-42, ch44-47, ch49-52; FDR-corrected, P = 0.000 ~ 0.028; Fig. 3). These channels were approximately located at bilateral superior frontal gyrus (SFG), bilateral middle frontal gyrus (MFG), bilateral inferior frontal gyrus (IFG), bilateral precentral gyrus, bilateral postcentral gyrus, bilateral supramarginal gyrus, bilateral superior temporal gyrus, and bilateral middle gyrus (Fig. 3). On the other hand, we found no significant differences in cortical activity over bilateral frontotemporal regions between the two groups during the CFT (see Supplementary Table S5 and S6 online).

### Correlational analyses

In the FES group, we did not find significant associations between DUP and cortical activities over frontotemporal regions during the LFT or CFT. No associations between DUP and LFT or CFT performances were found. In addition, there were no significant relationships between DUP and PANSS positive (rho: 0.28, n.s.), negative (rho: 0.31, n.s.) or general psychopathology scores (rho: 0.23, n.s.). With regard to the associations between antipsychotic dosage and VFT performance, no significant associations were found. On the other hand, longer DUP was significantly associated with poor SES (rho = 0.42, *P* = 0.02) (see Supplementary Table S7, S8, Fig. S1, and Fig. S2 online). In addition, we did not find any associations between cortical activities and duration of illness (DOI) (see Supplementary Table S9 and S10 online).

In FES patients, during the LFT, there was no significant correlation between mean [oxy-Hb] changes in any channel and PANSS positive scores (rho: −0.50 to 0.00, n.s.), PANSS general psychopathology scores (rho: −0.61 to 0.13, n.s.), or daily dosage level of antipsychotic drugs (rho: −0.33 to 0.55, n.s.), but a significantly negative correlation was observed between cortical activities and PANSS negative scores at 14 channels (ch20, ch30–33, ch40–45, ch50–52; rho = −0.71 to −0.48; FDR-corrected, P = 0.000 ~ 0.013). On the other hand, during the CFT, mean [oxy-Hb] changes were not significantly correlated with PANSS positive scores (rho: −0.33 to 0.23, n.s.), PANSS negative scores (rho: −0.52 to 0.34, n.s.) or daily dosage level of antipsychotic drugs (rho: −0.37 to 0.42, n.s.) in any channel, but a significantly negative correlation was observed with PANSS general psychopathology scores at 2 channels (ch20, ch30; rho: −0.67 to −0.63; FDR-corrected, P = 0.000 ~ 0.001).

## Discussion

Our results did not reveal an association between DUP and cortical activity over bilateral frontotemporal regions during either VFT in FES patients. To the best of our knowledge, this is the first study to use multichannel NIRS to investigate DUP effects on brain cortical activity during two versions of the VFT in FES patients. There main findings can be summarised as follows. (1) There were significant increases in both groups’ cortical activities over bilateral frontotemporal regions during both VFT. (2) There was a difference in the cortical activity patterns between the 2 groups during the LFT but not the CFT, indicating that deficits in cortical activity during phonemic processing may occur early in the course of SZ. (3) There was a lack of association between DUP and cortical activities during both VFTs.

### Increased cortical activity of frontotemporal regions during VFT

We found significantly increased cortical activities in the HC group during both types of VFT over bilateral frontotemporal regions, which is consistent with several previous NIRS studies^32^,^33^,^34^,^35^,^36^. However, during the LFT, we found significantly increased cortical activities over bilateral frontotemporal regions in the FES group, which is similar to the results reported by Takizawa *et al.* and Marumo *et al.* using 52 multi-channel NIRS^36^,^37^. However, Ehlis *et al.* did not show a significant increase in cortical activities over prefrontal regions^32^. With regard to CFT, the FES group showed significantly increased cortical activities over bilateral frontotemporal regions, which is similar to findings reported by Marumo *et al.*^37^, but again, inconsistent with those reported by Ehlis *et al.*^32^. These discrepancies may be attributable to at least two reasons. Firstly, Ehlis *et al.* used 22 channel NIRS whereas we used a 52-channel NIRS instrument that enabled us to examine cortical activities over a wider area. Secondly, these differences might be due to different participant characteristics. Variable patterns of cortical activity have been reported according to the progression of SZ clinical stages^34^. In the present study, we recruited SZ patients during the early stage of their illness, which is different from previous studies (mean duration of illness > 10 years)^32^,^33^,^35^,^37^.

### Group comparisons of NIRS signals and performance during the VFT

Our findings showed reduced cortical activities over bilateral frontotemporal regions during the LFT in the FES patients, which was consistent with previous NIRS studies focusing on FES^34^ as well as chronic SZ patients^32^,^33^,^35^,^36^,^38^. On the other hand, with regard to CFT, we did not find significant differences in cortical activities between FES and HC groups. Our results were consistent with those reported by Ikezawa *et al.*^33^, but inconsistent with those reported by others^32^,^35^,^37^. However, because those NIRS studies focusing on CFT recruited patients at a relatively chronic stage (mean illness duration > 10 years), it is difficult to directly compare the results. On the other hand, similar to previous studies^32^,^33^,^37^, group differences in cortical activations were more significant for the LFT than the CFT, indicating more pronounced phonological (letter fluency) deficits in FES patients^32^. Thus, it may suggest that cortical activities during the LFT may be a more sensitive indicator of frontal dysfunction in SZ than the CFT in SZ patients Taiwan.

With regard to the performance of VFTs, FES group scores lower in both types of VFT than HC group scores, which is consistent with the reports of a previous study^39^. Moreover, similar to previous NIRS studies^32^,^33^,^37^, we found that CFT performance was better than LFT in both groups. It was probably because compared to CFT, LFT is unfamiliar to subjects and more difficult^40^, and hence requires greater cognitive demands during LFT performance. This point may be supported by previous studies demonstrating that performance of CFT was better than that of LFT, but [oxy-Hb] changes during the LFT were greater than those during the CFT^32^,^33^. However, neuropsychological studies in Western populations have suggested that the category version of the VFT is more severely impaired than the letter version of the verbal fluency test in patients with schizophrenia^39^. Future studies designed to compare performance differences in the LFT and CFT and investigate the relationship with functional outcomes in Taiwanese SZ patients are warranted.

### Effects of DUP on brain cortical activity

We failed to provide evidence for a relationship between DUP and cognitive deficits or cortical activity changes during a VFT in FES patients. In a previous NIRS study investigating the effects of DUP on cortical activity, the authors recruited a group of SZ patients with duration of illness < 10 years^29^. They found no correlations between DUP and brain function in patients with SZ receiving antipsychotic treatment shorter than 6 months, which is similar to our results. We did not find a relationship between DUP and cortical activities in minimally treated FES patients. On the other hand, our findings are consistent with most previous neurocognitive^7^,^8^,^9^,^10^,^11^,^12^ and MRI studies^18^,^19^,^20^,^21^,^22^,^23^ focusing on this topic and confirms that there is little evidence for the neurotoxicity hypothesis. Indeed, our results appear to contradict the hypothesis that schizophrenia is a progressive disease. Alternatively, there is an extensive amount of empirical evidence that functional and structural brain changes occur before psychotic symptom onset^41^,^42^,^43^, suggesting that neurodevelopmental disturbances probably begin pre-morbidly and continue pro-dromally and after the onset of psychotic symptoms^24^. Therefore, changes in brain structure and cognitive function are independent of the effects of DUP^44^.

## Limitations

Our results should be viewed in light of several limitations. First, given the relatively small number of study participants, we may not have fully detected differences between groups; studies with larger samples and more detailed observation are needed. Second, selection bias must be considered as participants were recruited from the medical centre. Moreover, we had to consider the feasibility of taking measurements when recruiting, so potential selection bias according to participants’ symptoms and severities also needs to be considered. Third, because we employed a cross-sectional design, we could not examine longitudinal causal relationships between DUP and cortical activity. Finally, the effects of medication on cortical activity should also be considered. Although we recruited FES patients who were minimally treated with antipsychotic medication to reduce this confounding effect, recent studies showed that even short-term treatment with antipsychotics was associated with structural brain changes^45^. We also did not evaluate the effects of different types of antipsychotics used as most study participants received atypical antipsychotics. Future NIRS studies that assess a larger number of subjects with a focus on drug-naïve FES patients are warranted.

## Conclusion

In this study, we investigated the relationship between frontotemporal brain region activity and DUP in patients with FES by using two types of VFTs and 52-channel NIRS imaging. We found reduced cortical activities over bilateral frontotemporal regions in FES patients during the LFT but not CFT. Furthermore, we did not find an association between DUP and brain functions over frontotemporal regions. Our findings do not support to the neurotoxicity hypothesis. However, relationship between DUP and long-term clinical outcomes is well established, and there might be other consequences of delayed treatment, so these findings should not weaken the rationale for early detection and intervention strategies in SZ patients with a first psychotic episode.

## Methods

### Study participants

A total of 28 patients (15 males and 13 females) were recruited from the out- and inpatient populations at the Taichung Veterans General Hospital. Patients who met the criteria of Diagnostic and Statistical Manual of Mental Disorders, 4th edition, Text Revision (DSM-IV-TR)^46^ for SZ for the study were diagnosed by experienced psychiatrists (P.H.Chou, C.C.Lin, P.S. Hou, T.H. Lan, C.H. Chan, and C.C. Hung) and the diagnoses were validated using the Mini International Neuropsychiatric Interview (MINI)^47^. All patients were experiencing their first episode of psychosis and had received no more than 12 weeks of previous antipsychotic medication treatment^19^. Nine patients were taking risperidone, 8 paliperidone, 6 olanzapine, 3 aripiprazole, 1 amisulpride and 1 haloperidal.

Twenty-nine healthy individuals (10 males and 19 females) were recruited as control subjects and were screened with the MINI. All study participants were right-handed, which was defined as >70 points in the Edinburgh Inventory^48^. Subjects who had a history of substance abuse or dependence, mental retardation, neurological disorder, or a medical condition that could have affected brain structure or function were excluded. Controls were excluded if there was a personal history of any axis I or II disorder. This study complied with the Declaration of Helsinki, and was approved by the Institutional Review Board of Taichung Veterans General Hospital (approval No. CF13044). All participants received a complete explanation of the study and provided written informed consent.

### Clinical measurements

The Positive and Negative Syndrome Scale (PANSS)^49^ was used to assess symptoms on the same day as the NIRS measurements. DUP was defined as the time from psychosis onset until the start of adequate treatment. It was calculated from the time period between the onset of first psychotic symptoms and the initiation of antipsychotic treatment based on patient interview, corroborative history from family members, and medical records^50^. Socioeconomic status was assessed using the Hollingshead scale^51^. Patients’ antipsychotics are presented as chlorpromazine-equivalent doses^52^.

### Cognitive testing

Patients completed 160-s block-design VFTs (both letter and category version). We selected the VFT because previous studies have demonstrated deficits in brain cortical activity over bilateral frontotemporal regions by NIRS measurement in SZ patients during the VFT^34^,^36^,^37^,^53^,^54^,^55^. The 160-s block-design contains three different time periods: a 30-s pre-task period, a 60-s task period, and a 70-s post-task period. In the pre- and post-task periods, patients were instructed to fix their gaze at the centre of the screen and repeatedly count from one to five to control for and remove task-related motion artefacts. For the 60-s task period of the LFT, patients were instructed to say as many words as possible that started with a phonological syllable presented as an audible instruction by a computer. The task period comprised three continuous 20-s sub-periods, that were initiated by a single syllable selected from nine possible options (first, /(b)/, /(p)/, or /(d)/; second, /(t)/, /(l)/, or /(n)/; third, /(m)/, /(f)/,or /(dz)/). We chose these syllables because their frequencies of appearance at the beginning of Chinese words are moderate. In the CFT, subjects were asked to produce as many words as possible within a given semantic cue for 20 s each (first: ‘birds,’ ‘fish,’ or ‘insects’; second: ‘sweets,’ ‘fruits,’ or ‘vegetables’; third: ‘vehicles,’ ‘stationery items,’ or ‘home appliances’). Transitions between the 20-s sub-periods were immediate to encourage continuous performance. Before beginning each task session, subjects were given instructions on how to generate correct answers during the task periods by experienced researchers (P.H. Chou and W.H. Lin). Each subject performed three practice trials to ensure that they understood the instructions. We then recorded the total number of correct words generated during the task as an index of VFT performance.

### NIRS instrument

**Figure 4:**
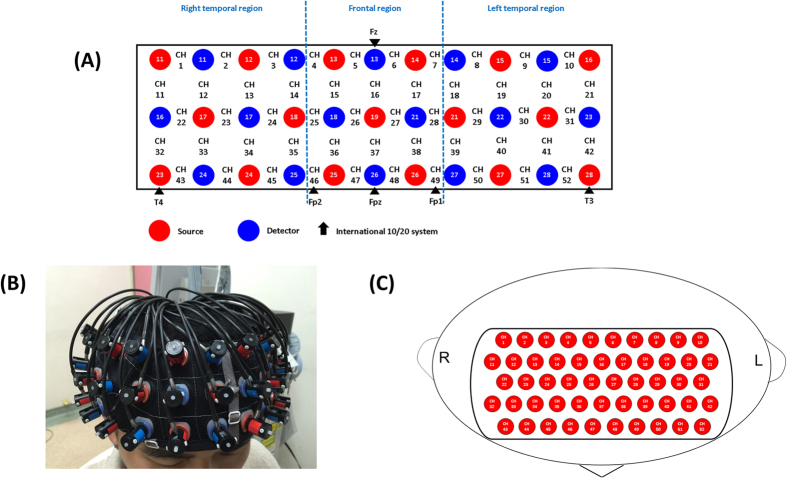
Probe setting and measurement points for 52-channel NIRS. (**A**) The localizations of all 52 channels were positioned according to the international 10–20 system. Red and blue circles indicate near-infrared light emitter and detector positions, respectively. By using the international 10–20 system, the detector 13 was positioned on the F z marker point, while the bottom row of channels was placed on a line between T 3 and T 4. (**B**) Probes with thermoplastic 3 × 11 shells were placed over bilateral frontotemporal regions. (**C**) The 52 measuring areas are labelled as ch1 to ch52 from the right posterior to left anterior.

Multichannel NIRS is a widely used functional neuroimaging technology that can measure the haemodynamics in the bilateral frontotemporal cortices. This technique enables spatiotemporal detection of brain function by measuring concentrations of oxy-haemoglobin ([oxy-Hb]) and deoxy-haemoglobin ([deoxy-Hb]), which reflect regional cerebral blood volume as demonstrated by their correlations with fMRI signals^56^. In the present study, we used a 52-channel NIRS instrument (ETG-4000; Hitachi Medical Co., Tokyo, Japan) to measure changes in [oxy-Hb]. The NIRS probe attachments are thermoplastic 3 × 11 shells set with 52 channels (Fig. 4). The lowest probe line was set along the Fp1–Fp2 line as defined by the international 10–20 system used in electroencephalography. The distance between pairs of source and detector probes was set to 3.0 cm. We defined the measurement area between each probe-set pair as one ‘channel,’ which was sufficient to measure depths between 20 and 30 mm under the scalp, approximately corresponding to the surface of the cerebral cortex.

NIRS measurements were performed with participants sitting in a chair with their eyes open and the probe attachment resting on the head. Participants were instructed to relax and avoid involuntary movements to minimize motion artifacts. The NIRS instrument measures changes in both [oxy-Hb] and [deoxy-Hb] by using two wavelengths (695 and 830 nm) of near-infrared light (indicated as mM) on the basis of the Beer–Lambert law^57^. We could not measure the absolute path length of each participant from the scalp to the cerebral cortex; therefore, we recorded the haemoglobin concentrations from baseline to activation periods. Relative changes in haemoglobin concentration assessed by NIRS measurements are indicated by mM·mm.

The data sampling rate was 0.1 s using the integral mode. The pre-task baseline was determined as the mean over a 10-s period immediately before the task period, and the post-task baseline was determined as the mean over the last 5 s of the post-task period. Linear fitting was applied to the data between these two baselines. A moving average method using a 5-s window width was applied and any short-term motion artefacts were rejected by an automatic artefact-rejection program in the NIRS instrument^38^. Because we excluded the rejected channels from further analysis, the number of available channels varied among individuals (LFT: FES group: 21–52 [mean, 47.6; SD, 6.9]; control group: 35–52 [mean, 48.1; SD, 5.2]; n.s.; CFT: FES group: 29–52 [mean, 47.8; SD, 5.8]; control group: 38–52 [mean, 48.4; SD, 4.4]; n.s).

The spatial information for each channel was estimated by using data from the Functional Brain Science Laboratory at Jichi Medical University in Japan (http://www.jichi.ac.jp/brainlab/virtual_reg.html);^58^ According to the LONI Probabilistic Brain Atlas (LPBA40)^59^, NIRS channels can record functional haemodynamics within the bilateral frontal, temporal, and parietal cortices. Similar to previous studies^29^,^38^,^53^, NIRS channels were anatomically labelled only after the LPBA region of highest probability was determined. We used the mean changes in [oxy-Hb] measured during the VFT as an index of brain cortical activity. We chose [oxy-Hb] as an indicator because it better reflects cortical activity and demonstrates stronger correlations with fMRI blood-oxygenation level-dependent signals compared to [deoxy-Hb]^56^.

## Statistical analysis

Each DUP value was transformed to the base 10 logarithm (log_10_ DUP) to manage skewness and moderate leverage data points^2^. Then, Spearman’s rank correlation coefficient was used to examine the relationship between log_10_ DUP and the mean [oxy-Hb] changes measured in each channel during the VFTs. The correction for multiple analyses among 52 channels was made using the false discovery rate method (FDR) (two-tailed; we set the value of q specifying the maximum FDR to 0.05, so that there are no more than 5% false positives on average)^60^. The correlation between DUP, LFT and CFT performance, and clinical parameters were also analyzed. Moreover, to detect any confounding factors, we also investigated the relationship between mean [oxy-Hb] changes and DOI, PANSS positive, negative, and general psychopathology scores, and daily dosage level of antipsychotic drugs using Spearman’s rank correlation coefficient. The effect of antipsychotic medication on performance of VFTs was also evaluated. Basic characteristics between groups and differences between LFT and CFT scores in each group were compared using Student’s t-tests and paired t-tests, respectively. All statistical analyses were performed with SPSS 18.0 software (IBM Inc., Armonk, NY, USA).

## Additional Information

**How to cite this article**: Chou, P.-H. *et al.* Duration of Untreated Psychosis and Brain Function during Verbal Fluency Testing in First-Episode Schizophrenia: A Near-Infrared Spectroscopy Study. *Sci. Rep.* **5**, 18069; doi: 10.1038/srep18069 (2015).

## Supplementary Material

Supplementary Information

Supplementary Dataset

## Figures and Tables

**Table 1:** Basic characteristics of study participants.

	**Schizophrenia group (n** **=** **28)**	**Healthy control group (n** **=** **29)**	**P value**
	Mean (SD)	Mean (SD)	
Age	30.8 (6.1)	30.3 (10.6)	0.80
Gender (Male/Female)^a^	(15/13)	(10/19)	0.19
Handedness (Right/Left)^b^	(28/0)	(29/0)	
SES	2.9(1.2)	2.6 (0.9)	0.33
Education (graduate/undergraduate/High school degrees)	(6/17/5)	(4/23/2)	0.28
LFT performance	8.9 (4.4)	12.8 (5.3)	0.00*
CFT performance	11.9 (4.2)	15.5 (4.7)	0.00*
DUP (week)	88.6 (119.7)		
DOI (week)	118.9 (130.7)		
Onset age	28.6 (6.1)		
PANSS			
Positive	16.5 (5.6)		
Negative	17.9 (6.0)		
General psychopathology	34.0 (9.3)		
Total	68.4 (16.8)		
Antipsychotic medication			
Chlorpromazine dosage	457.7 (220.8)		
Type (Typical/Atypical)	(1/27)		

Abbreviations: SES, Socioeconomic status; LFT, Letter version of Verbal Fluency Test; CFT, Category version of Verbal Fluency Test; DUP, Duration of Untreated Psychosis; DOI, Duration of Illness; PANSS, Positive and Negative Symptoms Scale.

^a^Chi-square test was used for testing group difference. Otherwise, t-tests were used.

^b^Right-handedness was defined as >70 points according to Oldfield’s Edinburgh Inventory.
