# Supplementary material for: Duration of Untreated Psychosis and Brain Function during Verbal Fluency Testing in First-Episode Schizophrenia: A Near-Infrared Spectroscopy Study
Source: Sci Rep. 2015 Dec 10;5:18069. doi: 10.1038/srep18069 (PMC4674798; doi:10.1038/srep18069)
Supplement: Supplementary Information [file srep18069-s1.pdf]

Duration of Untreated Psychosis and Brain Function during Verbal Fluency Testing in First-Episode Schizophrenia: A Near-Infrared Spectroscopy Study

Po-Han Chou, M.D; Wei-Hao Lin, M.D; Chih-Chien Lin, M.D., M.P.H.; Po-Shun Hou, M.D., M.S.; Wan-Rung Li, M.D.; Chia-Chun Hung, M.D.; Ching-Po Lin, Ph.D.;Tsuo-Hung Lan, M.D., Ph.D.; Chin-Hong Chan, M.D., M.S.

Supplementary Table S1.

Results of paired T-test showing significant increased [oxy-Hb] changes during letter version of verbal fluency test (LFT) in healthy control group.

No. of channels	Healthy controls (n=29)	
	t-value	*p* value[a]
CH01	5.085	0.000*
CH02	4.543	0.000*
CH03	5.011	0.000*
CH04	2.986	0.006*
CH05	3.523	0.002*
CH06	3.366	0.002*
CH07	2.551	0.018*
CH08	2.469	0.021*
CH09	5.946	0.000*
CH10	0.922	0.366
CH11	6.318	0.000*
CH12	5.046	0.000*
CH13	5.171	0.000*
CH14	7.017	0.000*
CH15	4.803	0.000*
CH16	4.493	0.000*
CH17	4.586	0.000*
CH18	5.402	0.000*
CH19	7.169	0.000*
CH20	5.378	0.000*
CH21	2.543	0.017*
CH22	6.493	0.000*
CH23	5.883	0.000*
CH24	8.756	0.000*
CH25	9.564	0.000*
CH26	8.206	0.000*
CH27	7.451	0.000*
CH28	6.633	0.000*
CH29	7.171	0.000*
CH30	6.007	0.000*
CH31	7.400	0.000*
CH32	7.609	0.000*

CH33	7.339	0.000*
CH34	5.743	0.000*
CH35	10.555	0.000*
CH36	9.505	0.000*
CH37	9.361	0.000*
CH38	7.201	0.000*
CH39	7.287	0.000*
CH40	6.127	0.000*
CH41	6.068	0.000*
CH42	6.464	0.000*
CH43	7.407	0.000*
CH44	7.214	0.000*
CH45	7.990	0.000*
CH46	10.291	0.000*
CH47	9.627	0.000*
CH48	6.534	0.000*
CH49	8.222	0.000*
CH50	9.278	0.000*
CH51	8.114	0.000*
CH52	8.118	0.000*

[a] Significance was defined as FDR corrected $p < 0.04904$.

Supplementary Table S2.

Results of paired T-test showing significant increased [oxy-Hb] changes during letter version of verbal fluency test (LFT) in schizophrenia group.

No. of channels	Schizophrenia (n=28)	
	t-value	*p* value[a]
CH01	2.207	0.036*
CH02	2.743	0.011*
CH03	2.191	0.037*
CH04	1.356	0.187*
CH05	0.985	0.334
CH06	0.929	0.362
CH07	0.810	0.426
CH08	2.047	0.052
CH09	2.777	0.010*
CH10	2.286	0.031*
CH11	3.093	0.005*
CH12	4.624	0.000*
CH13	3.033	0.006*
CH14	2.448	0.021*
CH15	1.107	0.279
CH16	1.660	0.109
CH17	2.466	0.021*
CH18	2.762	0.011*
CH19	4.060	0.000*
CH20	2.756	0.012*
CH21	2.625	0.015*
CH22	5.304	0.000*
CH23	4.014	0.001*
CH24	4.121	0.000*
CH25	2.816	0.009*
CH26	2.743	0.011*
CH27	2.221	0.035*
CH28	4.078	0.000*
CH29	4.688	0.000*
CH30	4.252	0.000*
CH31	3.822	0.001*
CH32	4.351	0.000*

CH33	4.459	0.000*
CH34	4.991	0.000*
CH35	3.423	0.002*
CH36	2.472	0.021*
CH37	2.993	0.006*
CH38	2.600	0.016*
CH39	3.928	0.001*
CH40	5.064	0.000*
CH41	5.111	0.000*
CH42	4.377	0.000*
CH43	2.078	0.050
CH44	4.181	0.000*
CH45	4.430	0.000*
CH46	3.190	0.004*
CH47	3.231	0.004*
CH48	2.915	0.008*
CH49	2.864	0.008*
CH50	4.980	0.000*
CH51	4.950	0.000*
CH52	4.156	0.000*

[a] Significance was defined as FDR corrected $p < 0.04327$.

Supplementary Table S3.

Results of paired T-test showing significant increased [oxy-Hb] changes during category version of verbal fluency test (CFT) in healthy control group.

No. of channels	Healthy control (n=29)	
	t-value	*p* value[a]
CH01	3.573	0.001*
CH02	3.399	0.002*
CH03	1.762	0.093
CH04	2.306	0.030*
CH05	2.529	0.018*
CH06	2.460	0.022*
CH07	0.245	0.808
CH08	0.919	0.368
CH09	1.106	0.279
CH10	3.298	0.003*
CH11	3.794	0.001*
CH12	4.817	0.000*
CH13	5.134	0.000*
CH14	4.261	0.000*
CH15	3.708	0.001*
CH16	2.764	0.010*
CH17	1.557	0.132
CH18	2.639	0.014*
CH19	5.090	0.000*
CH20	5.623	0.000*
CH21	5.087	0.000*
CH22	4.869	0.000*
CH23	5.829	0.000*
CH24	6.415	0.000*
CH25	6.043	0.000*
CH26	4.660	0.000*
CH27	4.758	0.000*
CH28	5.043	0.000*
CH29	6.339	0.000*
CH30	4.771	0.000*
CH31	5.257	0.000*
CH32	5.788	0.000*

CH33	5.948	0.000*
CH34	6.118	0.000*
CH35	7.567	0.000*
CH36	4.947	0.000*
CH37	4.022	0.000*
CH38	4.492	0.000*
CH39	7.470	0.000*
CH40	5.951	0.000*
CH41	5.721	0.000*
CH42	5.833	0.000*
CH43	6.706	0.000*
CH44	7.049	0.000*
CH45	8.036	0.000*
CH46	7.641	0.000*
CH47	4.768	0.000*
CH48	4.675	0.000*
CH49	6.108	0.000*
CH50	7.452	0.000*
CH51	6.712	0.000*
CH52	6.437	0.000*

[a] Significance was defined as FDR corrected $p < 0.04519$.

Supplementary Table S4.

Results of paired T-test showing significant increased [oxy-Hb] changes during category version of verbal fluency test (CFT) in schizophrenia group.

No. of channels	Schizophrenia (n=28)	
	t-value	*p* value[a]
CH01	-0.016	0.987
CH02	-1.050	0.304
CH03	-0.973	0.339
CH04	0.384	0.704
CH05	0.325	0.748
CH06	1.326	0.198
CH07	0.902	0.375
CH08	0.001	1.000
CH09	1.923	0.068
CH10	0.901	0.377
CH11	2.476	0.020*
CH12	2.042	0.051
CH13	3.364	0.002*
CH14	1.497	0.146
CH15	0.674	0.507
CH16	1.370	0.183
CH17	1.362	0.185
CH18	0.589	0.561
CH19	1.100	0.282
CH20	2.963	0.007*
CH21	1.773	0.088
CH22	3.081	0.005*
CH23	3.294	0.003*
CH24	4.151	0.000*
CH25	2.465	0.020*
CH26	2.336	0.029*
CH27	1.987	0.058
CH28	1.985	0.058
CH29	3.261	0.003*
CH30	3.759	0.001*
CH31	4.228	0.000*
CH32	3.662	0.001*

CH33	3.530	0.002*
CH34	3.241	0.003*
CH35	4.075	0.000*
CH36	2.490	0.020*
CH37	2.640	0.014*
CH38	2.253	0.034
CH39	3.133	0.005*
CH40	3.582	0.001*
CH41	4.095	0.000*
CH42	3.979	0.001*
CH43	3.624	0.001*
CH44	4.076	0.000*
CH45	3.945	0.001*
CH46	2.990	0.006*
CH47	2.380	0.026*
CH48	2.954	0.008*
CH49	2.894	0.008*
CH50	5.042	0.000*
CH51	3.801	0.001*
CH52	4.276	0.000*

[a] Significance was defined as FDR corrected $p < 0.02981$.

Supplementary Table S5.

Results of T-test showing significantly higher [oxy-Hb] changes during letter version of verbal fluency test (LFT) in healthy control group than schizophrenia group.

No. of channels	*t-value*	*p* value[a]
CH01	2.873	0.006*
CH02	1.450	0.153
CH03	1.469	0.148
CH04	0.988	0.327
CH05	2.098	0.041
CH06	2.043	0.046
CH07	1.663	0.104
CH08	0.964	0.339
CH09	2.316	0.025*
CH10	-0.697	0.489
CH11	3.588	0.001*
CH12	1.638	0.108
CH13	1.314	0.195
CH14	1.535	0.131
CH15	2.363	0.022*
CH16	1.759	0.084
CH17	1.859	0.069
CH18	2.180	0.034
CH19	2.254	0.028*
CH20	2.279	0.027*
CH21	0.253	0.801
CH22	2.679	0.010*
CH23	1.826	0.075
CH24	2.511	0.015*
CH25	2.634	0.011*
CH26	3.180	0.002*
CH27	2.792	0.007*
CH28	1.901	0.063
CH29	1.906	0.062
CH30	2.391	0.022*
CH31	2.880	0.006*

CH32	3.749	0.000*
CH33	2.373	0.021*
CH34	2.431	0.020*
CH35	2.996	0.004*
CH36	3.361	0.001*
CH37	1.241	0.220
CH38	1.662	0.103
CH39	2.281	0.027*
CH40	2.441	0.018*
CH41	2.545	0.014*
CH42	2.803	0.007*
CH43	0.220	0.826
CH44	2.834	0.007*
CH45	3.444	0.001*
CH46	2.918	0.005*
CH47	2.297	0.026*
CH48	1.884	0.065
CH49	2.555	0.014*
CH50	2.708	0.009*
CH51	3.482	0.001*
CH52	3.365	0.001*

[a] Significance was defined as FDR corrected $p < 0.02885$.

Supplementary Table S6.

Results of T-test showing significant increased [oxy-Hb] changes during category version of verbal fluency test (CFT) between healthy control and schizophrenia group.

No. of channels	*t-value*	*p* value[a]
CH01	3.076	0.004
CH02	1.580	0.120
CH03	0.234	0.816
CH04	2.096	0.041
CH05	2.078	0.043
CH06	1.033	0.307
CH07	0.901	0.372
CH08	0.674	0.503
CH09	-0.198	0.843
CH10	1.207	0.238
CH11	1.743	0.087
CH12	2.119	0.039
CH13	0.171	0.866
CH14	1.675	0.100
CH15	2.024	0.048
CH16	0.920	0.361
CH17	0.241	0.810
CH18	1.665	0.102
CH19	2.408	0.020
CH20	1.167	0.249
CH21	2.314	0.025
CH22	1.538	0.130
CH23	1.497	0.141
CH24	1.814	0.075
CH25	1.967	0.055
CH26	1.422	0.161
CH27	1.898	0.063
CH28	1.454	0.152
CH29	1.812	0.076
CH30	0.941	0.352
CH31	1.770	0.083

CH32	2.516	0.015
CH33	1.430	0.158
CH34	1.861	0.068
CH35	1.843	0.072
CH36	1.746	0.087
CH37	0.166	0.869
CH38	0.565	0.574
CH39	1.516	0.136
CH40	2.869	0.007
CH41	1.248	0.218
CH42	2.398	0.020
CH43	2.478	0.017
CH44	1.834	0.073
CH45	2.328	0.024
CH46	1.953	0.056
CH47	0.914	0.365
CH48	0.592	0.562
CH49	1.644	0.112
CH50	2.717	0.009
CH51	2.462	0.017
CH52	1.458	0.150

[a] Significance was defined as FDR corrected $p < 0.00096$.

Supplementary Table S7.

Results of Spearman's rank correlation between [oxy-Hb] changes during letter version of verbal fluency test (LFT) and DUP.

No. of channels	*rho*	*p* value[a]
CH01	-0.276	0.163
CH02	0.032	0.876
CH03	0.051	0.796
CH04	-0.193	0.334
CH05	-0.284	0.144
CH06	-0.151	0.462
CH07	-0.038	0.861
CH08	-0.188	0.367
CH09	0.262	0.196
CH10	0.037	0.859
CH11	0.039	0.842
CH12	-0.132	0.521
CH13	-0.086	0.670
CH14	-0.088	0.658
CH15	-0.225	0.280
CH16	-0.153	0.436
CH17	-0.267	0.187
CH18	-0.302	0.133
CH19	-0.442	0.027
CH20	-0.239	0.272
CH21	-0.289	0.170
CH22	-0.108	0.598
CH23	-0.308	0.175
CH24	-0.197	0.325
CH25	-0.101	0.615
CH26	-0.198	0.312
CH27	-0.316	0.101
CH28	-0.329	0.094
CH29	-0.417	0.030
CH30	-0.162	0.507
CH31	-0.123	0.576
CH32	-0.310	0.140
CH33	-0.218	0.284

CH34	-0.302	0.151
CH35	-0.012	0.954
CH36	-0.103	0.617
CH37	0.077	0.709
CH38	-0.208	0.319
CH39	-0.238	0.241
CH40	-0.404	0.041
CH41	-0.063	0.759
CH42	-0.332	0.113
CH43	-0.278	0.170
CH44	-0.307	0.144
CH45	-0.187	0.380
CH46	0.094	0.642
CH47	0.123	0.558
CH48	0.223	0.284
CH49	0.106	0.606
CH50	-0.146	0.477
CH51	-0.023	0.910
CH52	0.097	0.669

[a] Significance was defined as FDR corrected $p < 0.00096$.

Supplementary Table S8.
Results of Spearman's rank correlation between [oxy-Hb] changes during category version of verbal fluency test (CFT) and DUP.

No. of channels	*rho*	*p* value[a]
CH01	-0.218	0.275
CH02	-0.097	0.636
CH03	-0.007	0.970
CH04	0.033	0.868
CH05	0.031	0.880
CH06	-0.187	0.382
CH07	0.239	0.240
CH08	0.366	0.072
CH09	0.144	0.511
CH10	0.132	0.528
CH11	0.095	0.639
CH12	-0.139	0.480
CH13	-0.155	0.442
CH14	0.112	0.571
CH15	-0.182	0.384
CH16	0.046	0.823
CH17	-0.163	0.417
CH18	-0.044	0.831
CH19	-0.005	0.980
CH20	-0.278	0.199
CH21	-0.383	0.054
CH22	-0.031	0.880
CH23	-0.222	0.287
CH24	-0.068	0.731
CH25	0.024	0.903
CH26	0.211	0.323
CH27	0.036	0.858
CH28	-0.142	0.488
CH29	-0.076	0.714
CH30	-0.234	0.294
CH31	-0.299	0.156
CH32	-0.065	0.752

CH33	-0.112	0.578
CH34	-0.185	0.366
CH35	0.015	0.942
CH36	0.120	0.568
CH37	0.295	0.152
CH38	-0.110	0.599
CH39	-0.132	0.531
CH40	-0.127	0.537
CH41	-0.030	0.887
CH42	-0.260	0.200
CH43	-0.091	0.666
CH44	-0.229	0.282
CH45	-0.084	0.678
CH46	0.116	0.574
CH47	0.268	0.206
CH48	0.198	0.376
CH49	0.162	0.460
CH50	-0.146	0.467
CH51	-0.132	0.511
CH52	-0.101	0.616

[a] Significance was defined as FDR corrected $p < 0.00096$.

Supplementary Table S9.

Results of Spearman's rank correlation between [oxy-Hb] changes during letter version of verbal fluency test (LFT) and DOI.[a]

No. of channels	*rho*	*p* value[b]
CH01	-0.302	0.125
CH02	-0.068	0.742
CH03	-0.037	0.853
CH04	-0.343	0.080
CH05	-0.395	0.038
CH06	-0.280	0.165
CH07	-0.186	0.385
CH08	-0.271	0.190
CH09	0.191	0.349
CH10	-0.016	0.940
CH11	-0.059	0.766
CH12	-0.241	0.235
CH13	-0.141	0.484
CH14	-0.203	0.301
CH15	-0.375	0.065
CH16	-0.307	0.112
CH17	-0.394	0.046
CH18	-0.412	0.036
CH19	-0.493	0.012
CH20	-0.309	0.151
CH21	-0.390	0.060
CH22	-0.176	0.390
CH23	-0.307	0.175
CH24	-0.249	0.210
CH25	-0.216	0.280
CH26	-0.350	0.068
CH27	-0.407	0.031
CH28	-0.462	0.015
CH29	-0.514	0.006
CH30	-0.314	0.190
CH31	-0.318	0.139
CH32	-0.384	0.064
CH33	-0.269	0.184

CH34	-0.317	0.131
CH35	-0.111	0.580
CH36	-0.251	0.217
CH37	-0.104	0.612
CH38	-0.275	0.183
CH39	-0.335	0.095
CH40	-0.526	0.006
CH41	-0.085	0.679
CH42	-0.481	0.017
CH43	-0.319	0.112
CH44	-0.422	0.040
CH45	-0.283	0.179
CH46	-0.049	0.808
CH47	-0.061	0.773
CH48	0.049	0.815
CH49	0.046	0.823
CH50	-0.276	0.172
CH51	-0.094	0.648
CH52	-0.016	0.942

[a]Each DOI value was transformed to the base 10 logarithm (log DOI)

[b]Significance was defined as FDR corrected $p < 0.00096$.

Supplementary Table S10.

Results of Spearman's rank correlation between [oxy-Hb] changes during category version of verbal fluency test (CFT) and DOI.

No. of channels	*rho*	*p* value[a]
CH01	-0.301	0.127
CH02	-0.153	0.457
CH03	-0.007	0.970
CH04	-0.048	0.808
CH05	-0.065	0.748
CH06	-0.186	0.384
CH07	0.292	0.147
CH08	0.304	0.139
CH09	-0.009	0.968
CH10	0.043	0.837
CH11	0.041	0.839
CH12	-0.189	0.335
CH13	-0.191	0.339
CH14	0.033	0.866
CH15	-0.307	0.136
CH16	-0.043	0.833
CH17	-0.168	0.402
CH18	-0.094	0.649
CH19	-0.134	0.513
CH20	-0.376	0.077
CH21	-0.500	0.009
CH22	-0.151	0.461
CH23	-0.244	0.239
CH24	-0.143	0.468
CH25	-0.072	0.716
CH26	0.052	0.810
CH27	-0.100	0.620
CH28	-0.214	0.295
CH29	-0.200	0.328
CH30	-0.412	0.057
CH31	-0.412	0.046
CH32	-0.215	0.291
CH33	-0.190	0.342

CH34	-0.259	0.202
CH35	-0.098	0.626
CH36	-0.021	0.921
CH37	0.155	0.460
CH38	-0.240	0.248
CH39	-0.255	0.218
CH40	-0.213	0.295
CH41	-0.071	0.735
CH42	-0.365	0.067
CH43	-0.242	0.244
CH44	-0.312	0.137
CH45	-0.152	0.449
CH46	0.018	0.930
CH47	0.169	0.429
CH48	0.083	0.713
CH49	0.059	0.788
CH50	-0.317	0.107
CH51	-0.136	0.499
CH52	-0.176	0.380

[a]Each DOI value was transformed to the base 10 logarithm (log DOI)

[b]Significance was defined as FDR corrected $p < 0.00096$.

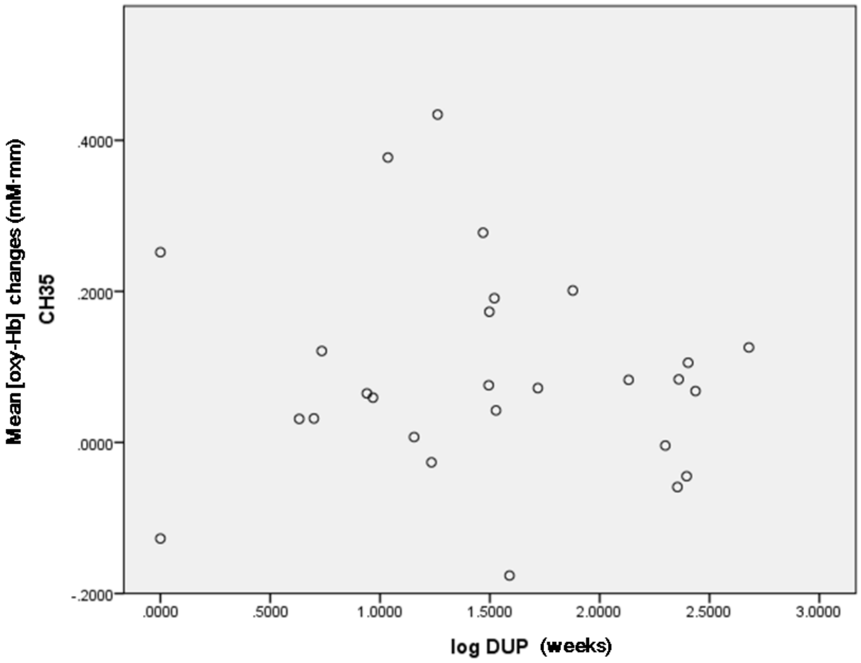

Supplementary Figure S1. This figure illustrates correlations between the mean [oxy-Hb] changes during LFT and DUP in FES patients.The scatter plot illustrates typical nonsignificant channel in ch35. (Spearman's rank correlation coefficient rho = -0.01, n.s.)

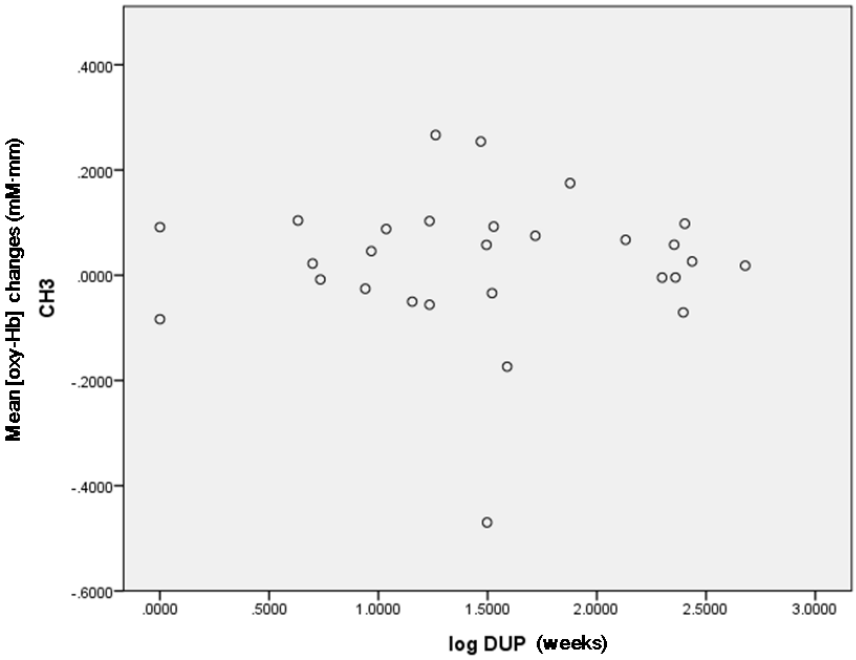

Supplementary Figure S2. This figure illustrates correlations between the mean [oxy-Hb] changes during CFT and DUP in FES patients. The scatter plot illustrates typical nonsignificant channel in ch3. (Spearman's rank correlation coefficient rho = -0.007, n.s.)
